# 

**Published:** 1853-11

**Authors:** 


*Books Received.*

FROM BLANCHARD & LEA—Miller's Practice of Surgery; Wilde on Diseases of the Ear; Taylor's Medical Jurisprudence; Budd on the Liver; Williams' Principles of Medicine; Condie on Diseases of Children.

FROM LINDSAY & BLAKISTON—Walton's Ophthalmic Surgery; Headland on the Action of Medicines; Henle's General Pathology.

All of which will be noticed in our next.

### RECEIPTS

### FOR THE JOURNAL DURING THE MONTHS OF OCTOBER AND NOVEMBER.

### ILLINOIS.

| | |
|---|---|
| Dr. G. W. Kittelle, | $2 00 |
| " C. W. Knott, | 2 00 |
| " Robinson, | 4 00 |
| " H. S. Strong, | 4 00 |
| " Boynton Tenney, | 2 00 |
| " J. B. Tenney, | 2 00 |
| " P. D. Miller. | 1 50 |
| " Martin & Michner, | 2 00 |
| " Roger, | 4 00 |
| " Helm, | 4 00 |
| " H A. Raney, | 2 00 |
| " A. Hagar, | 2 00 |
| " A. Hard, | 2 00 |
| " E. A. Rogers, | 2 00 |
| " J. H. Rider, | 1 00 |
| " H C. Donaldson, | 2 00 |
| " Bodenstat, | 2 00 |
| " A, Atherton, | 2 00 |
| " Daniel Wood, | 4 00 |
| " M. Davis, | 2 00 |
| " N S. Anderson, | 2 00 |
| " N. English, | 2 00 |
| " O. Long, | 4 00 |
| " H. D. Grant, | 4 00 |
| " D Smith, | 2 00 |
| " A. P. Finch, | 2 00 |
| " Willis Danforth, | 2 00 |
| " Griswold, | 2 00 |
| " Seth Sogier, | 4 00 |
| " P. W, Ranelle, | 2 00 |
| " G. P. Ransom, | 2 00 |
| " Chas Irwin, | 4 00 |
| " B. Harris, | 1 00 |
| " Snowdon, | 2 00 |
| " Hess, | 4 00 |
| " G, W. Albin, | 2 00 |
| " G, W. P. Harding, | 6 00 |
| " A Sumner Haskill | 2 00 |
| " J W Spaulding, | 2 00 |
| " B S Robinson, | 4 00 |
| " E Woodruff, | 4 00 |
| " Thompson Meade, | 2 00 |
| " Wm T Briscoe, | 2 00 |
| " N B Butler, | 2 00 |

### OHIO

| | |
|---|---|
| Dr. J Fisher, | $4 00 |
| " S Stough | 2 00 |
| " Hazlitt, | 2 00 |

### WISCONSIN.

| | |
|---|---|
| Dr. Thos Steel, | $2 00 |
| " John L Scofield, | 2 00 |
| " L B W Johnson, | 2 00 |
| " A P Adams, | 2 00 |
| " E P Wood, | 4 00 |
| " J R Goodnough | 2 00 |
| " J H Morrison, | 2 00 |
| " R W Earll, | 2 00 |

### INDIANA.

| | |
|---|---|
| Dr. Kelsey, | $4 00 |
| " B F Russell | 4 00 |
| " J F McCarthy, | 2 00 |
| " Lewis H Sovereign, | 2 00 |
| " R C Bond, | 2 00 |
| " John S Thompson, | 2 00 |
| " R C Pierce. | 2 00 |
| " C W Davis | 2 00 |
| " W Brenton, | 2 00 |
| " E H Sutton, | 2 00 |
| " L E Carver, | 4 00 |
| " Luther Jewett | 2 00 |
| " N B Clausen | 2 00 |
| " Clay Brown. | 2 00 |
| " Wm Farhurst, | 2 00 |
| " A Wood, | 1 00 |
| " D Newell, | 2 00 |

### IOWA

| | |
|---|---|
| Dr. A B Ireland, | $3 00 |
| " W D Nelson, | 2 00 |
| " H Fisk | 1 00 |
| " J N B Elliott, | 2 00 |
| " Watson, | 2 00 |
| " E Hollingsworth, | 2 00 |

### MICHIGAN.

| | |
|---|---|
| Dr. Brayton, | $4 00 |
| " J B W Lewis, | 2 00 |
| " W H Elliot, | 9 00 |
| " A Lewis, | 1 00 |
| " R S Hawley, | 2 00 |
| " J M'Lean, | 2 00 |

### MISSOURI.

| | |
|---|---|
| Dr. E C Still, | $2 00 |
| " E D M'Gorrisk | 2 00 |

### ALABAMA.

| | |
|---|---|
| Dr. R E Haughton, | $2 00 |

